# Longitudinal Pancreaticojejunostomy Reconstruction Following Pancreaticoduodenectomy for Patients With Concomitant Chronic Pancreatitis: How I Do It

**DOI:** 10.1002/ags3.70098

**Published:** 2025-10-20

**Authors:** Hideaki Sato, Masaharu Ishida, Naoki Rikiyama, Masamichi Mizuma, Michiaki Unno

**Affiliations:** ^1^ Department of Surgery Tohoku University Graduate School of Medicine Sendai Japan

**Keywords:** chronic pancreatitis, lateral pancreaticojejunostomy, longitudinal pancreaticojejunostomy, pancreaticoduodenectomy

## Abstract

Surgical management of pancreatic head lesions complicated by chronic pancreatitis (CP) presents significant challenges, particularly in ensuring effective pancreatic duct drainage after resection. This report describes the detailed technique and key considerations for longitudinal pancreaticojejunostomy (LPJ) as a reconstructive method following pancreaticoduodenectomy (PD) in patients with CP. This approach aims to achieve wide‐area drainage of the remnant pancreatic duct through a side‐to‐side anastomosis between the extensively opened pancreatic duct and jejunum. Conceptually derived from conventional drainage procedures such as the Frey and Partington procedures, this technique is particularly beneficial when significant ductal dilatation persists distal to the resection margin. Critical steps for successful LPJ include meticulous exposure of the pancreatic parenchyma, an adequate longitudinal incision of the main pancreatic duct, and precise anastomotic technique. We have successfully applied this procedure in three male patients (median age 68 years, range: 48–77), including one with CP complicated by duodenal bleeding and two with pancreatic head region malignancies complicated with CP. All patients had favorable postoperative outcomes with no clinically relevant postoperative pancreatic fistula (Grade B or C). Long‐term follow‐up showed significant symptom improvement without medication, and pancreatic function were relatively well preserved. Our preliminary experience suggests that LPJ provides safe and effective drainage of the pancreas following PD in patients with CP‐complicated pancreatic head pathology and may represent a valuable reconstructive option, particularly in cases with marked pancreatic duct dilatation.

## Graphical abstract

This report details a modified reconstructive technique, longitudinal pancreaticojejunostomy (LPJ), following pancreaticoduodenectomy (PD) for patients with both pancreatic head pathology and extensive chronic pancreatitis. This approach ensures wide‐area drainage of the remnant pancreatic duct, providing effective symptom relief and preserving long‐term pancreatic function, as demonstrated in three successful cases. Our experience suggests that LPJ is a valuable and safe option for selected patients

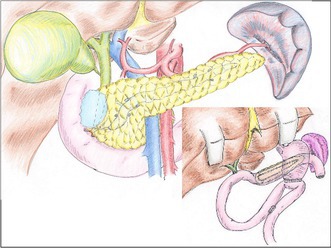

## 1 Introduction

The primary objectives of surgical management for chronic pancreatitis (CP) are to relieve intractable abdominal pain, manage exocrine and endocrine insufficiency, and prevent disease‐related complications [1]. Surgical strategies are generally categorized into two main approaches, each based on distinct therapeutic principles: pancreatic duct drainage and pancreatic resection. Drainage procedures include pancreatojejunostomy with distal pancreatectomy (the Puestow procedure) [2], longitudinal pancreaticojejunostomy (LPJ), also known as the Partington procedure [3]. While resectional procedures comprise distal pancreatectomy (DP), pancreatoduodenectomy (PD), and duodenum‐preserving pancreatic head resection (the Beger procedure) [4]. The Frey procedure, a LPJ with coring‐out of the pancreatic head, theoretically combines elements of both drainage and resection, and is also commonly employed [5, 6].

The optimal surgical strategy for CP is determined by the patient's anatomical and pathological features. In cases involving extensive pancreatic head disease, particularly with duodenal involvement or concomitant malignancy, PD is often indicated. However, in selected cases, especially when significant ductal dilatation persists due to intraductal calculi distal to the resected segment of PD, a combined approach involving both resection and drainage may be required to achieve sufficient ductal decompression and symptom relief.

At our institution, we have adopted a modified reconstructive approach incorporating LPJ following PD in selected patients with CP. This strategy is conceptually based on conventional procedures such as the Frey and Partington procedures [3, 5, 6]. In this report, we describe the technical details and clinical rationale for LPJ reconstruction following PD in three patients: one with CP complicated by duodenal involvement and gastrointestinal hemorrhage, and two others with duodenal cancer and distal cholangiocarcinoma, respectively, both also complicated by CP. The underlying cause of CP in all three cases was alcohol.

## 2 Surgical Technique

### 2.1 Ethics

The present study protocol was approved by the Ethics Committee of Tohoku University (approval number: 2025‐1‐239).

### 2.2 Indications

LPJ reconstruction following PD is selectively indicated for patients with concomitant CP who require surgical resection. Our specific indications include CP cases complicated by extensive duodenal inflammation, resulting in hemorrhage or stricture. This approach is also considered for patients undergoing PD for pancreatic head malignancies (e.g., duodenal cancer, distal cholangiocarcinoma, or pancreatic head cancer) who have concomitant dilatation of the main pancreatic duct due to an intraductal obstruction in the remnant pancreas. A critical criterion for this procedure is significant ductal dilation and pathological changes (fibrosis, strictures, and intraductal stones) extending into the pancreatic body and tail. In such cases, postoperative decompression of the remaining pancreatic duct is essential due to CP‐related ductal pathology in the pancreas distal to the resection margin. LPJ serves to ensure adequate drainage of the pancreatic body and tail, thereby contributing to pain relief and preservation of residual pancreatic function.

### 2.3 Operative Procedures

**FIGURE 1:**
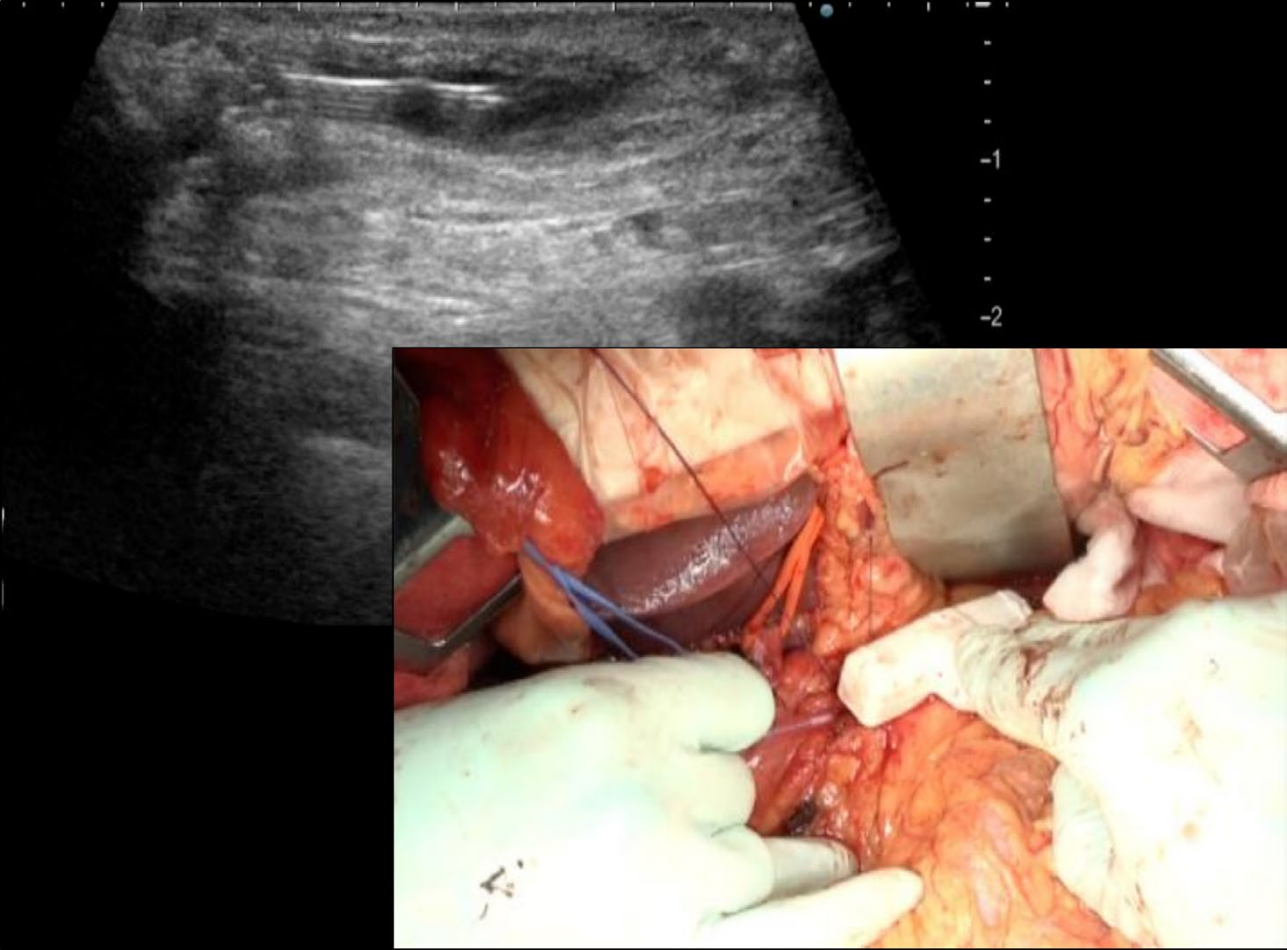
Intraoperative ultrasound‐guided puncture of the main pancreatic duct. The main pancreatic duct is being identified and punctured under real‐time ultrasound guidance to facilitate safe and accurate localization prior to longitudinal incision.

First, a standard PD is performed [7]. After resection, the pancreatic remnant and main duct are carefully assessed. The cut surface is meticulously prepared to allow for wide exposure of the main pancreatic duct. Given the fibrotic nature of CP, delicate dissection is essential to avoid parenchymal injury. When the main duct is identifiable at the cut surface, a longitudinal incision is made distally toward the pancreatic tail. If identification is challenging, intraoperative ultrasound is used to guide ductal puncture and facilitate safe longitudinal opening (Figure 1) (Video S1).

**FIGURE 2:**
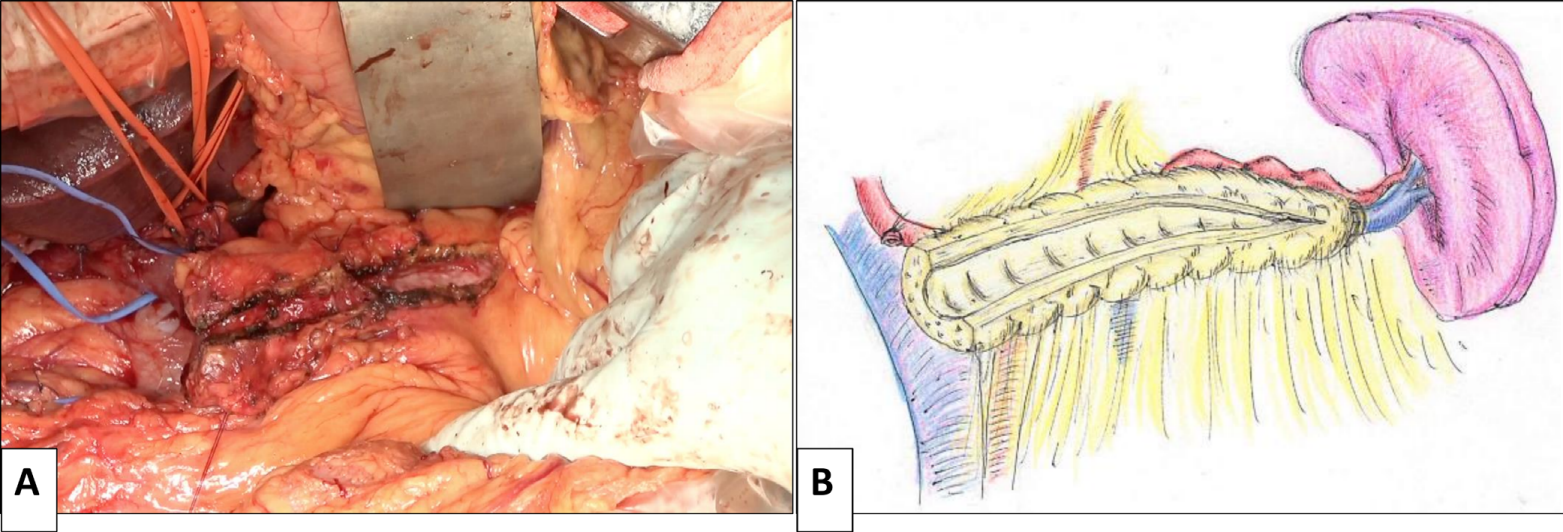
Longitudinal incision of the main pancreatic duct in the pancreatic remnant. The dilated main pancreatic duct is incised longitudinally toward the pancreatic tail to allow for adequate drainage, while carefully preserving surrounding parenchyma. (A) Intraoperative photograph. (B) Schematic illustration.

A wide longitudinal incision of the main duct is essential for effective LPJ. The duct is incised distally as far as feasible, and any visible intraductal stones are removed (Video S1). Care is taken to remain within the ductal wall and avoid excessive damage to the surrounding parenchyma (Figure 2A,B).

**FIGURE 3:**
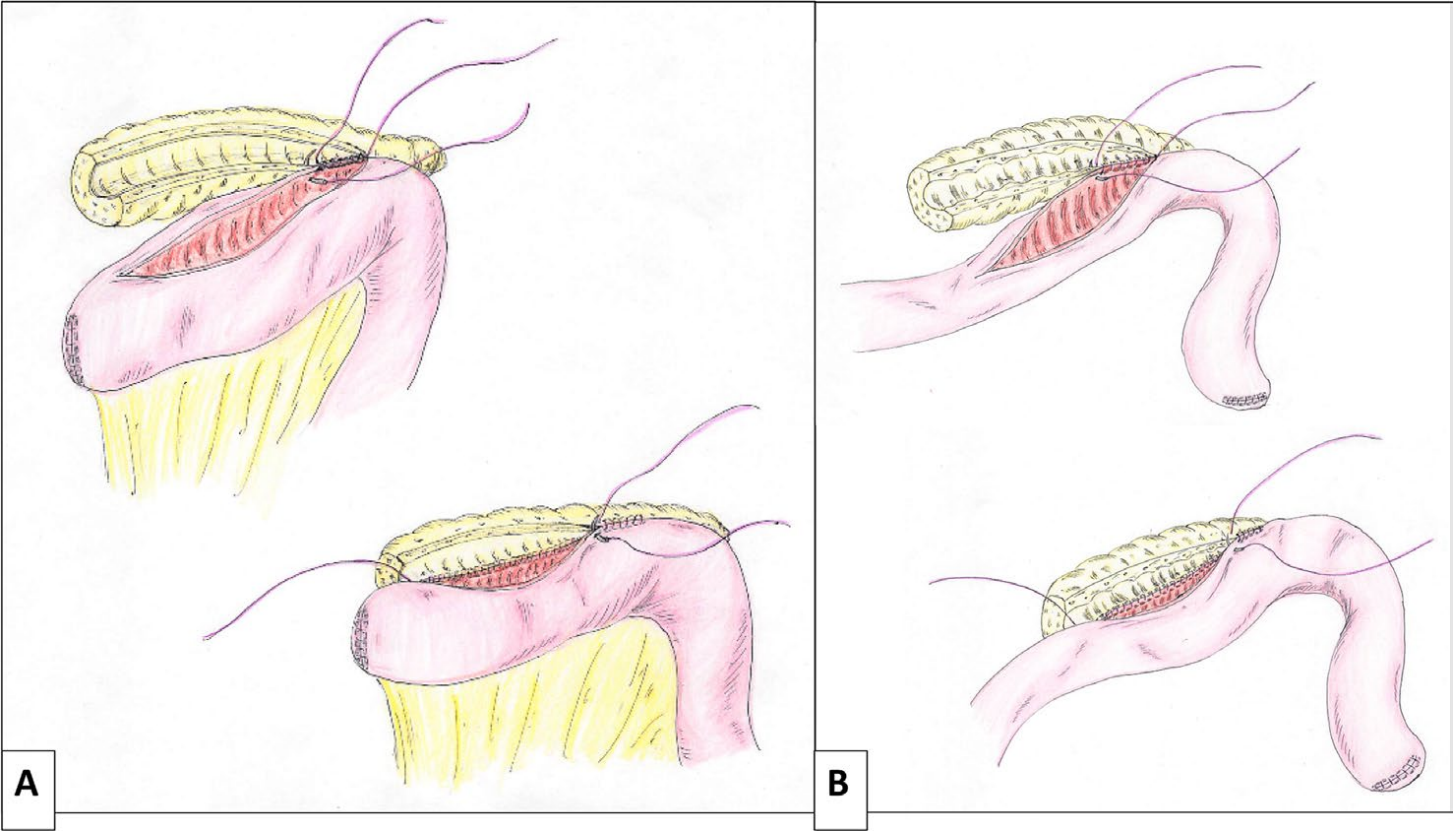
Construction of the longitudinal pancreaticojejunostomy (LPJ). LPJ is performed using a single‐layer continuous suture technique with 4‐0 non‐absorbable monofilament. The posterior wall of the anastomosis is completed first, followed by the anterior wall. (A) The jejunal limb is positioned toward the pancreatic head (Case 1). (B) The jejunal limb is positioned toward the pancreatic tail (Cases 2 and 3).

The stump of the jejunum is prepared and brought to the pancreas via a retrocolic route. LPJ is constructed using a single‐layer continuous suture technique with 4‐0 non‐absorbable monofilament. The posterior wall is sutured first, followed by the anterior wall (Figure 3A,B). A pancreatic duct stent is not routinely inserted (Video S2).

**FIGURE 4:**
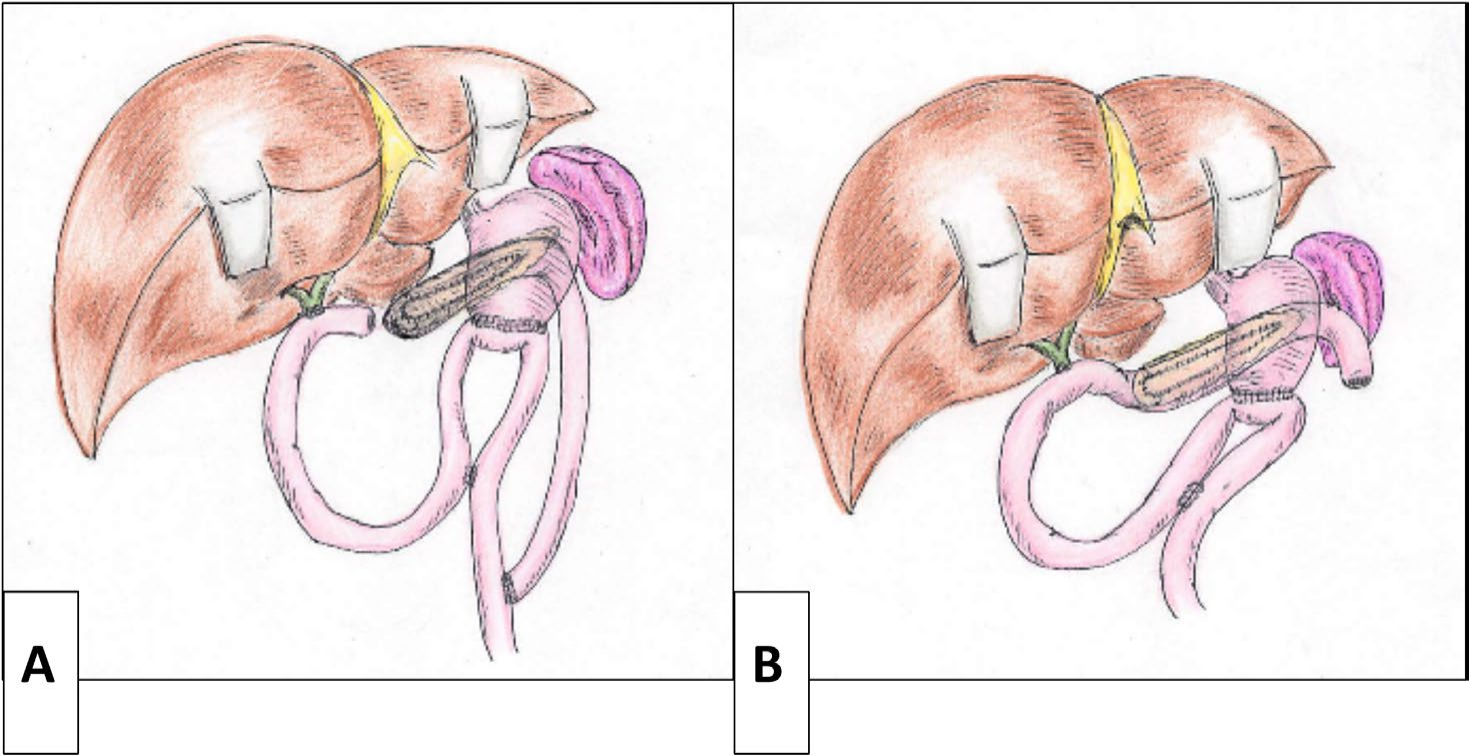
Schematic diagrams of the reconstructive procedures. (A) A Roux‐en‐Y jejunal limb is used for both pancreaticojejunostomy and hepaticojejunostomy (Case 1). (B) Reconstruction is performed using the modified child method (Cases 2 and 3).

Following LPJ, hepaticojejunostomy and gastrojejunostomy are performed using standard techniques. Reconstruction is performed using either a Roux‐en‐Y jejunal limb that serves for hepaticojejunostomy (Case 1) (Figure 4A) or the modified Child method (Cases 2 and 3) (Figure 4B). Closed‐suction drains are placed near the anastomosis to monitor for postoperative pancreatic fistula (POPF).

## 3 Results

We successfully performed LPJ as a reconstructive method following PD in three patients with CP requiring surgical resection. The demographic characteristics, operative details, and postoperative outcomes for each patient are summarized below.

### 3.1 Patient Demographics and Preoperative Status

All three patients were male, with a median age of 68 years (range: 48–77 years). The indications for PD were: CP with severe duodenal involvement and gastrointestinal bleeding in Case 1; duodenal cancer in Case 2; and distal cholangiocarcinoma complicated by CP in Case 3 (Table 1).

**TABLE 1:** Patient characteristics of all three cases.

No	Age (year)	Sex (M:F)	Cause of PD	Operative time (min)	Blood loss (mL)	POPF	Postoperative hospital stay (day)
Case 1	48	M	Duodenal bleeding	735	2320	None	25
Case 2	68	M	Duodenal cancer	457	1527	None	20
Case 3	77	M	Distal cholangiocarcinoma	448	465	BL	20

### 3.2 Operative Details

All three patients successfully underwent PD with LPJ. The mean operative time was 457 min (range: 448–735 min), and the mean estimated blood loss was 1527 mL (range: 465–2320 mL). No intraoperative complications related to the LPJ procedure were observed. In all cases, the remnant main pancreatic duct was significantly dilated, allowing for a longitudinal incision to be made without difficulty. Side‐to‐side anastomosis between the opened pancreatic duct and the jejunal limb was completed smoothly. Pancreatic duct stents were not routinely used in these cases.

### 3.3 Postoperative Course and Complications

All three patients had an uneventful postoperative course without major surgical complications. Importantly, no clinically relevant postoperative pancreatic fistula (POPF) (Grade B or C, as per the ISGPS classification [8]) occurred in any case. One patient (Case 3) developed a minor POPF (Grade BL), which resolved spontaneously with conservative management. The mean postoperative hospital stay was 20 days (range: 20–25 days). No other postoperative complications were observed such as postoperative bleeding, intra‐abdominal abscess, or anastomotic stricture.

### 3.4 Long‐Term Outcomes and Follow‐Up

Patients were followed for a median period of 78 months (range: 3–130 months). All three patients reported a significant improvement in CP‐related symptoms, particularly the resolution of abdominal pain. Pain relief was assessed through patient interviews and defined as a complete resolution or significant reduction in the frequency and intensity of pain, and none required medication for pain relief. No recurrence of gastrointestinal bleeding was observed in Case 1. Among the two patients with malignancies (Cases 2 and 3), oncological outcomes were consistent with their disease stages. Case 2 remains disease‐free 78 months postoperatively, and Case 3 showed no evidence of recurrence at 3 months after surgery. Regarding pancreatic function, none of the patients developed new‐onset diabetes mellitus postoperatively, and pre‐existing diabetes in Case 3 remained well controlled without deterioration. With regard to exocrine function, although all patients required pancreatic enzyme replacement therapy to support digestion as a consequence of PD, their nutritional status a year after surgery was relatively well maintained, as reflected by stable serum albumin levels and body weight (from a preoperative average 3.4 g/dL to postoperative average 4.0 g/dL and from 51.8 to 47.1 kg, respectively). Follow‐up CT imaging demonstrated a patent LPJ with good drainage of the remnant pancreatic duct in all cases, confirming both the anatomical and functional success of the reconstruction.

## 4 Discussion

Surgical strategies for CP are generally categorized into two main strategies: drainage and resectional procedures [1, 9]. While resection is often necessary in cases involving bleeding or malignancy, a major concern remains the potential deterioration of the remaining pancreatic function [1, 10]. Inadequate drainage of the remaining pancreatic duct following resection may lead to continued progression of CP, resulting in persistent abdominal pain and further impairment of both exocrine and endocrine function. Conversely, numerous reports suggest the benefits of drainage procedures including effective pain relief and improved nutritional status [11, 12]. Therefore, securing adequate drainage of the remnant pancreatic duct is essential in CP patients undergoing PD.

The rationale for our combined approach is to address both the tumor and the diffuse pathology of obstructive CP in a single procedure. While PD eliminates the pancreatic head‐often regarded as the “pain pacemaker”‐this alone is insufficient in cases with extensive calcification and multiple strictures extending into the body and tail. Without the addition of LPJ, further drainage procedures would likely have been required to achieve durable pain relief and preserve pancreatic function.

Based on these principles, we have adopted the concurrent application of PD with LPJ in selected cases. Our extensive experience with the Frey procedure has informed and supported this approach [6, 11, 12, 13]. Previous studies have also reported the utility of LPJ in treating pancreaticojejunostomy strictures after PD and its potential to improve glucose tolerance [14, 15]. The combination of LPJ with PD has been reported as an effective strategy to avoid overly extensive pancreatic resection [16, 17], thereby contributing to the preservation of postoperative pancreatic function. In our presented cases, this combined approach proved both rational and effective in minimizing the extent of resection while preserving long‐term pancreatic function.

Our patients experienced favorable perioperative and long‐term outcomes. A major concern following PD is the risk of POPF. In our three cases, while the fibrotic parenchyma typically seen in CP reduces the risk of POPF, no clinically relevant POPF was observed. Additionally, long‐term follow‐up demonstrated the maintenance of both glucose tolerance and nutritional status. These postoperative benefits, similar to those of the Frey procedure [12], were achieved by preserving the pancreatic body and tail.

While the limited number of cases precludes broad generalizations, it is worth noting that combination procedures are well‐established in pancreatic surgery for CP [6, 13, 18, 19]. Examples include Frey plus distal pancreatectomy (Frey + DP) [6, 13] and Frey plus choledochojejunostomy (Frey + CJ) [18, 19]. Consequently, combining PD with LPJ aligns well with current surgical philosophy and practice. Given the theoretical foundation and favorable outcomes, we propose that this combined approach is a viable and meaningful option for selected patients with CP.

## 5 Conclusions

LPJ represents a viable and potentially superior reconstructive option following PD for selected patients with CP requiring resection.

## Author Contributions

**Hideaki Sato:** conceptualization, methodology, data curation, investigation, validation, formal analysis, writing – original draft, project administration, visualization. **Masaharu Ishida:** supervision, writing – review and editing, writing – original draft, project administration, conceptualization, validation, investigation. **Naoki Rikiyama:** visualization, writing – review and editing. **Masamichi Mizuma:** writing – review and editing, conceptualization, methodology, validation, supervision, project administration. **Michiaki Unno:** writing – review and editing, conceptualization, supervision, project administration, methodology, validation.

## Ethics Statement

The study protocol was conducted in accordance with the ethical standards outlined in the Declaration of Helsinki and was approved by the Institutional Review Board of Tohoku University (approval number: 2025‐1‐239).

## Consent

Written informed consent was obtained from all participants regarding the use of their clinical data.

## Conflicts of Interest

The authors declare no conflicts of interest.

## Supporting information


**Video S1:** Intraoperative ultrasound is used to guide ductal puncture and facilitate safe longitudinal incision.

**Video S2:** Longitudinal pancreaticojejunostomy (LPJ) is constructed using a single‐layer, continuous suture with 4‐0 non‐absorbable monofilament.
